# An innovative assessment tool for evaluating narrative feedback quality among Medicine and Biomedical Sciences students

**DOI:** 10.5116/ijme.64f6.df43

**Published:** 2023-10-12

**Authors:** Michelle M.J. Jacobs, Pauline M. van Son, Alwin Scharstuhl, Petra J. van Gurp, Esther Tanck

**Affiliations:** 1 Radboudumc Health Academy, Nijmegen, the Netherlands; 2 Department of Internal medicine, Radboudumc, Nijmegen, the Netherlands; 3 Department of Orthopaedics, Radboudumc, Nijmegen, the Netherlands

**Keywords:** Feedback education, feedback quality, assessment, feedback assessment instruments, rubric

## Abstract

**Objectives:**

To develop a reliable instrument to objectively assess feedback quality, to use it for assessment of the quality of students’ narrative feedback and to be used as a self-assessment instrument for students in their learning process.

**Methods:**

In a retrospective cohort study, 635 feedback narratives, provided by small groups of Medicine and Biomedical Sciences undergraduate students, have been extracted from available quarterly curriculum evaluation surveys. A rubric was developed based on literature and contents of our feedback education. It consists of seven subitems and has a maximum score of 20 points (sufficient score: >10 points). Rubric reliability was evaluated using intra-class correlation. The rubric was tested by analysing the feedback narratives. To test progression, we compared rubric scores between study years with a Kruskal-Wallis analysis and Dunn’s post-hoc testing with Bonferroni correction.

**Results:**

The rubric has an intra-class correlation of 0.894. First year students had a mean rubric score of 11.5 points (SD 3.6), second year students 12.4 (SD 3.4) and third year students 13.1 (SD 3.6). Kruskal-Wallis testing showed significant differences in feedback quality between study years (χ^2^(2, N=635) = 17.53, p<0.001). Dunn’s post-hoc test revealed significant differences between study years one and two (p=0.012) and one and three (p<0.001).

**Conclusions:**

The developed rubric is a reliable instrument to assess narrative feedback quality. Students were able to provide feedback of sufficient quality and quality improved across study years. The instrument will allow students to assess themselves and learn where there is still room for improvement.

## Introduction

Feedback has been valuable for numerous purposes like students’ learning process and improving education.^1^ It has been used extensively in the academic setting where students receive feedback from teachers and peers in order to improve their learning process. Moreover, students have regularly been asked to give feedback to their teachers and the curriculum, to improve the quality of education.^2^^,^^3^

To convey the message and to be effective, feedback must be of good quality and thus it is a logical step to teach students how to provide effective feedback. Effective feedback consists of several components.^4^^-^^6^ Feedback preferably starts with an opinion about a certain performance. The next step is called ‘feed-up’, which is a description of the desired performance. Feed-up is followed by ‘feedback plus’: an objective explanation of the opinion. Lastly, feedback should be concluded with suggestions for improvement or an action plan, which is called ‘feedforward’.^6^ These components are also referred to as task (opinion), gap (feed-up and feedback plus) and action (feedforward).^7^  Moreover, feedback needs to be specific and well substantiated by giving arguments to empower the feedback.^6^^, ^^8^ It is important to deliver feedback in a neutral or non-judgemental way; this ensures the receiver not to feel attacked.

At the Radboud University of Nijmegen, the three-year Bachelor of Science (undergraduate) curricula of Medicine and Biomedical Sciences (focussed on biomedical research) share a common trunk considering professional development and skills training.^9^ Students learn how to request, receive and give constructive feedback. We have embedded an assignment that invites students to provide feedback as part of an evaluation cycle of their medical education. In short, students were regularly asked to provide feedback to one of the curriculum courses using so-called thematic surveys. The surveys consisted of five closed questions that were individually answered, and one open question that was answered after a discussion in a small group of students. After analysing the feedback, course coordinators completed the evaluation cycle by writing a statement for students outlining action points, based on the received feedback, to improve the quality of education. In this way a win-win situation was created for both the course coordinators and students. So, in the course ‘Professionalism’, students were trained to give feedback, to discuss the narrative group feedback, and to formulate constructive feedback voluntarily. However, whether their feedback was of sufficient quality and whether there was progression in feedback quality over the years is as yet unknown. As students’ feedback is used frequently (e.g., as peer feedback or to teachers for curriculum improvement), its quality needs to be high to ensure optimal feedback uptake.

Effective use of student feedback has been investigated extensively and it has been shown that students’ feedback is helpful in improving education quality.^2^^,^^10^^,^^11^ Particularly, student feedback literacy has received much attention.^12^ Feedback literacy concerns the understanding of feedback processes and the capacity to manage affect and to take action in response to feedback.^13^ However, the quality of feedback given by students has scarcely been studied. Wu and Schunn (2020)^14^ have looked at determinants of peer feedback uptake by students and used feedback quality as one parameter. However, their results on feedback quality were not reported descriptively. Gaynor (2020)^15^ assessed peer feedback quality with three assessment criteria but did not define norms for high- and poor-quality feedback. It thus remains unclear how well students are able to provide good quality feedback.

The quality of feedback may not only depend on the provider’s feedback giving skills, but also on the affect between the feedback provider and feedback recipient. Adams (2005)^16^ found that less positive and less specific feedback was given to liked individuals compared to disliked individuals. Highly liked courses might thus receive less specific feedback, which is of lower quality than specific feedback. We therefore questioned whether differences in program appreciation were of influence on the quality of feedback given by students. Moreover, not all aspects of giving feedback receive an equal amount of attention during the feedback education. It remains unknown on which feedback aspects students perform well or poorly. This knowledge could help in further improving education on feedback, by focussing more on aspects on which students perform poorly.

To assess feedback quality, an instrument to do so is required. Such an instrument would provide teachers and students insight in the level of feedback quality and on which aspects they can improve. Despite the availability of previously developed instruments^7^^,^^14^^,^^17^^-^^20^ it remains difficult to objectively measure feedback quality. Disadvantages of available instruments are that they were too specific to adopt to other curricula^17^^,^^19^ or feedback quality was solely based on the presence of feedback components (task, gap, action)^7^^,^^18^^,^^20^ or a complex scoring system was used which reduces applicability.^14^ There is a need for an effective widely applicable instrument that is able to measure feedback quality objectively, taking into account numerous aspects of feedback, and is able to promote improvement of feedback skills.

In this study, our primary goal was to develop a reliable, effective and useful instrument for objective self-assessment of written narrative feedback quality. Using this instrument, we aimed to analyse the quality of narrative feedback provided by Medicine and Biomedical Sciences undergraduate students. We tested whether the quality of narrative feedback improved over the three undergraduate study years. We hypothesised that the quality of feedback narratives of third-year students will be better than that of first-year students, as third-year students have had more education and have more often practiced giving feedback to peers and teachers than first-year students. Moreover, we studied more in-depth on which rubric subitems students performed well and we studied the influence of students’ course appreciation on the quality of feedback in general.

## Methods

### Study design and participants

A retrospective cohort study was performed to test our newly developed instrument that measures feedback quality. The cohort consisted of first, second- and third-year students of the undergraduate curricula of Medicine (n=1030 students (65% females)) and Biomedical Sciences (n=281 students (69% females)) at the Radboud university medical centre in Nijmegen, the Netherlands. Except for ten Medicine students and one Biomedical Sciences student, all students had the Dutch nationality.

The Dutch Association for Medical Education (NVMO) granted ethical approval for the conduct of this study. Given the design of the study, they did not consider additional ethical review or participant consent to be required.

### Instrument development

We developed a rubric that is tailored to the content of feedback education over the three undergraduate academic years and based on current literature on rubric development and feedback theory (Table 1).^21^ Only items addressed in our educational program were incorporated in the rubric, as using items unfamiliar to students would produce unreliable results in the assessment of narratives. A rubric was chosen as the preferred method because it is known to help researchers score as objectively as possible.^21^ First, we analysed our first year feedback educational course in order to formulate

**Table 1:** Newly developed rubric to measure feedback quality

Subitem	Insufficient		Sufficient		Good		Excellent	
	Points	Explanation	Points	Explanation	Points	Explanation	Points	Explanation
Usability	0	Feedback does not contain any of the required elements*	2	Feedback contains 1 element	4	Feedback contains 2 elements	6	Feedback contains all 3 elements
Context	0	Feedback is not clear and does not contain examples	2	Feedback is clear, but does not contain examples or has not been placed into context	4	Feedback is clear and examples are present, and/or context is clear		
Structure	0	Structure is absent*	1	Feedback elements are present, but not in the correct order*	2	Correct structure*		
Applicability	0	Feedback is not applicable	1	Feedback is partially applicable	2	Feedback is applicable next study year		
Answer to the question	0	Feedback does not answer the feedback question	1	Feedback partly answers the feedback question	2	Feedback fully answers the feedback question		
Language	0	Feedback answer is offensive towards the program	1	Feedback answer is neutral	2	Feedback is written as an “I-message”		
Spelling and grammar	0	≥ 3 spelling and grammar mistakes	1	1-3 mistakes and grammar errors, or use of half sentences	2	No mistakes in spelling and grammar		

Each feedback answer is scored on all seven subitems. The maximum number of points per feedback answer is 20. Items “usability” and “context” had a maximum score of 6 respectively 4 points, while other items had a maximum score of 2 points. *Feedback elements were: opinion, argumentation and a suggestion for improvement. This was also the correct order to receive 2 points for structure.

Each feedback answer is scored on all seven subitems. The maximum number of points per feedback answer is 20. Items “usability” and “context” had a maximum score of 6 respectively 4 points, while other items had a maximum score of 2 points. *Feedback elements were: opinion, argumentation and a suggestion for improvement. This was also the correct order to receive 2 points for structure.

potential rubric subitems. Next, we performed a literature review in order to substantiate rubric items. In a calibration session all authors agreed on seven subitems: usability^6^^,^^22^^-^^24^ (feedback containing three elements: opinion, argumentation and suggestion for improvement), context^25^, structure^6^ applicability^22^^,^^24^ answer to the raised question^22^^,^^26^ language^24^ and spelling and grammar^14^ (Table 1). The subitems were formulated as specific as possible. A pilot run was performed, where two researchers (M.J. and P.S.) independently scored 50 random feedback narratives. Results were compared, and discrepancies and needs for changes in formulation of subitems were discussed. This procedure was repeated with an improved rubric. After three cycles consensus among researchers was reached on definition and interpretation of the subitems. Rubric reliability was evaluated after scoring using intra-class correlation (ICC).

Total rubric score was calculated by adding up the individual subitem scores (Table 1). Subitems usability and context were deemed the most important factors of effective feedback according to literature^17^ and also received most attention during feedback training. These subitems therefore received double weighting. The range of total rubric score lies between zero and twenty points, with twenty points being the perfect score. We defined feedback with a score below 11 points as insufficient, feedback with 11-14 points as sufficient and feedback with 15 or more points as good. These cut-off points were based on the Dutch grading system, where students need to score 55% to pass and 75% to get a ‘good’ as grade.

### Data collection and study setting

The described cohort of students trained feedback skills in small, fixed groups of approximately eight students, guided by a coach.^9^ First year students have learned the theoretical background of giving and receiving feedback. They learned to value the importance of feedback and to provide others with effective feedback. Giving and receiving peer feedback was practised with each other in the context of working together on group projects. Second and third year students have focussed on extending their feedback literacy. While they kept practising with providing (and receiving) feedback, they were also expected to increasingly ask feedback on several themes of professionalism, to reflect on it and to apply the feedback if suited. As described in the introduction, all students were regularly invited to give narrative feedback to curriculum courses through thematic surveys. In group meetings, students discussed their feedback content and formulated as a group written narrative feedback to coordinators of the course. This is both part of training feedback skills and a means of evaluating and improving the curriculum. The coach was not involved in formulating the feedback, but may guide the process if deemed necessary. Narrative feedback was sent to the curriculum organisation via online survey software (LimeSurvey).

All fourteen thematic surveys conducted in study year 2018/2019 were retrospectively used for data collection. Feedback narratives from these surveys were obtained from the faculty evaluation service in 2020 by a member of our research team (A.S.). Five surveys were from the first study year, six from the second study year and three from the third study year. These surveys yielded a total of 641 group responses to open questions. Of all 641 responses, six answers were excluded because they did not contain an answer to the open question (e.g., “My classmate has already filled out this survey.”), leaving 635 responses for analysis, of which 251 in study year 1, 287 in study year 2 and 96 in study year 3. Two authors (M.J. and P.S.) independently scored all feedback narratives with the rubric. In case the total rubric scores differed less than four points, the lowest score of the two researchers was used for analysis. In case the difference was higher, the scoring was discussed in a consensus meeting with four authors (M.J., P.S., A.S., E.T.; n=84). Consensus was reached on all 84 narratives.

### Data analysis

#### Bias control

In order to minimise the risk of information bias during scoring, the mention of study year was removed from the feedback narratives, and all other clues that could reveal students’ study year were also removed. While these measures blinded most of the answers, very few narrative feedback could still have given a clue to the study year of the students. This may have led to subconscious information bias in researchers. To account for any undesired bias, an independent researcher who had no connections with the professionalism course and who was not involved with the study design and rubric development scored 10% (n=63) of all responses. One-way ANOVA was used to compare these scores with the scores of the researchers to determine the presence of information bias.

Being part of the curriculum might affect the interpretation of data. By assembling the research group with representation from students, faculty and educational organization, we assume that no single value or assumption can prevail. By collecting and interpreting data repeatedly, in a transparent process for the entire research group and recurrent reflecting to correct for own values and views, we avoided bias.

### Feedback quality

We performed descriptive analysis of our results to obtain the average rubric score per study year. To study if a statistical difference between quality of feedback existed between study years, we first tested data for normality between study years using the Kolmogorov-Smirnov test. Data was not normally distributed in all study years (D_(252) _= 0.086, p<0.001 for study year 1; D_(287)_ = 0.090, p<0.001 for study year 2 and D_(96)_ = 0.124, p<0.001 for study year 3), hence the Kruskal-Wallis H test and Dunn’s post-hoc test with Bonferroni correction were used to analyse differences in total rubric scores between study years. We used the scores from the consensus meeting and, in case of answers with less than four points difference between researchers, the lowest given score of the two researchers. The analysis was repeated with the highest given scores to test for any different outcomes. Statistical analyses were performed with SPSS version 24 software. Figures were created with the ggplot2 package in R. Statistical significance was set at α≤0.05.

Feedback quality might be influenced by students’ affect towards the feedback recipient.^16^ To analyse if there was a correlation between course appreciation and the quality of narrative feedback given by students, course appreciation grades per study year were obtained from general surveys from the faculty and correlated to mean rubric score per course with a one-tailed bivariate Pearson correlation test.

To analyse on which aspects of giving feedback students performed well or poorly, we also analysed the rubric subitem scores individually. All scores (n=635 per subitem) were averaged and the mean percentage scores for each subitem were calculated, to analyse on which subitems students scored insufficient (<55%), sufficient (55-75%) or good (>75%).

## Results

### Bias control

Analysis of all 635 feedback answers showed a rubric reliability with an ICC of 0.894. Bias control using the 63 feedback answers scored by the independent researcher showed that the two allocated blinded researchers and the independent blinded researcher scored similar overall and also per study year. Of all years combined, researchers one, two and three gave mean scores of 12.8 (SD = 3.8), 12.9 (SD = 4.1) and 12.2 (SD = 4.0), respectively, which did not differ significantly according to the performed one-way ANOVA test (F_(2, 180)_ = 0.660, p=0.518).

### Feedback quality

In general, the quality of narrative feedback was sufficient with a mean score of 12.1 points (SD = 3.6). More in detail, each study year scored on average a sufficient grade. Study year one had a mean score of 11.5 points (SD = 3.6), study year two 12.4 points (SD = 3.4) and study year three 13.1 points (SD = 3.7). The proportion of feedback narratives with a sufficient or good score was 63% (41% sufficient, 22% good) in study year one, 72% (42% sufficient, 30% good) in study year two and 76% (40% sufficient, 36% good) in study year three. A Kruskal-Wallis H test showed significant differences in feedback quality scores between study years (χ^2^ (2, N = 635) = 17.53, p<0.001) with a mean rank score of 284.6 for study year one, 329.8 for study year two and 370.4 for study year three. Dunn’s post hoc test with Bonferroni correction revealed statistically significant differences between study years one and two (p=0.012) and one and three (p<0.001).

Study years two and three did not differ significantly from each other (p=0.178). The same analysis but performed with the highest rubric scores given by the two researchers, was not different from the one described above (performed with lowest rubric scores), as the result of the Kruskal-Wallis H test was χ^2^ (2, N=635)=13.73, p=0.001 with a mean rank score of 289.1 for study year one, 327.2 for study year two and 366.3 for study year three. The results of the Dunn’s post-hoc test again showed statistically significant differences between study years one and two, and one and three (p=0.47 and p=0.001, respectively), but no significant difference between study years two and three (p=0.208).

To illustrate the difference between high- and poor-quality feedback, we translated one example of each from Dutch to English. These feedback answers are displayed in Table 2, together with their scores for the different subitems and total scores.

**Table 2:** Examples of high- and poor-quality feedback (translated from Dutch to English) with their rubric subitem and total scores

Quality	Feedback	Subitem/total	Points
High quality feedback	“We would appreciate an answer model that explains why certain answers are correct or wrong. We can currently only see which answer is correct and we think that it is way more informative to receive an explanation with a reference to the study material. This will help us better understand our mistakes.”	Usability	6
		Context	4
		Structure	1
		Applicability	1
		Answer to the question	2
		Language	2
		Spelling and grammar	2
		Total	18
Poor quality feedback	“Give better and more in-depth lectures!!!”	Usability	2
		Context	0
		Structure	0
		Applicability	0
		Answer to the question	1
		Language	0
		Spelling and grammar	1
		Total	4

**Figure 1:**
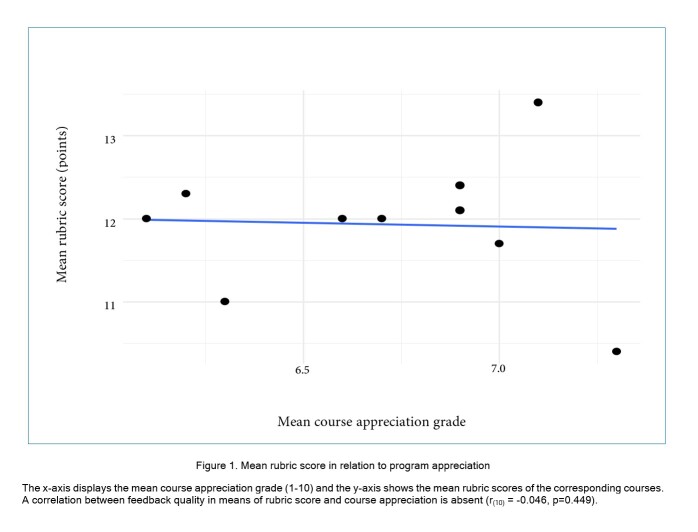
Mean rubric score in relation to program appreciation

To analyse whether a correlation between feedback quality and course appreciation exists, we compared the program appreciation scores given by students in the general surveys with rubric scores in our thematic surveys. The mean students’ appreciation for all courses was 6.71 (SD = 0.41). There was no correlation between quality of narrative feedback and program appreciation (r_(10)_= -0.046, p=0.449)(Figure 1).

The mean percentage score per subitem of the rubric showed that students scored a good grade on subitem answer to the question, a sufficient grade on the subitems usability, context and language, and an insufficient grade on the subitems structure, applicability, and spelling and grammar (Table 3).

## Discussion

In the absence of an instrument to measure quality of narrative feedback in the medical education context, we described the development of an instrument to objectively assess feedback quality. We tested it by evaluating the quality of written narrative feedback of small groups of Medicine and Biomedical Sciences students throughout the undergraduate curricula in the context of training the feedback skills of these students. Our study showed that the instrument is reliable for assessing feedback quality. Moreover, we showed that in the context of a training program for feedback skills, narrative feedback skills of all students were on average of sufficient quality and improved over the years. In this study, higher program appreciation did not affect feedback quality substantially, in contrast to the study by Adams (2005).^16^ Hence, our findings indicate that feedback education and the connection with ‘real world’ assignments offers students opportunities to develop the necessary skills to properly give feedback.

**Table 3:** Average rubric scores per subitem (n=635 feedback narratives)

Item (maximum score (points))	Mean score (points) (SD)	% of maximum score
Usability (6)	4.4 (1.5)	72.8
Context (4)	2.7 (1.2)	67.8
Structure (2)	0.8 (0.3)	42.4*
Applicability (2)	1.1 (0.8)	53.0*
Answer to the question (2)	1.8 (0.4)	91.4
Language (2)	1.1 (0.4)	56.3
Spelling and grammar (2)	0.8 (0.6)	37.9*

SD: standard deviation; *: insufficient grade, defined as a mean score

### Rubric for assessment of feedback quality

We created a novel instrument for objective feedback quality assessment, as existing instruments did not match our requirements. Previously, Gauthier and colleagues (2015)^7^ and Abraham and Singaram (2019)^20^ developed simple and easy to use rubrics. They based feedback quality solely on the presence and content of the feedback components: task, gap and action. These instruments lack other factors that determine quality, such as applicability^22^^, ^^24^ and polite use of language.^24^ Our rubric hence assesses feedback on all relevant determinants of feedback quality. Warm and colleagues (2018)^19^ developed a feedback assessment instrument for the rating of feedback given by faculty members. This instrument is not applicable in a feedback training setting for students. We aimed to develop a rubric that is widely applicable with neutrally formulated criteria within a general context. Our instrument is a useful addition to the currently available Feedback assessment instruments as it was proven to be reliable, measures feedback quality as objectively as possible, uses multiple determinants of feedback quality, is easy to use for self-assessment and can hence be utilised by others.

### Feedback quality

On average, students provided feedback of sufficient quality (defined as a rubric score of 11 points or higher), with 70% of all feedback comments scoring sufficient or good. Gaynor (2020)^15^ investigated peer feedback quality on the items of being specific or constructive and being helpful for the receiver. That study found that 50-60% of feedback scripts were coded as specific or constructive and 83-86% of students found the received peer feedback helpful. Although our results are difficult to compare on scored items but were studied in similar contexts of training feedback skills, it seems clear that in both studies more than half of the students gave feedback of sufficient quality or feedback that was deemed useful. This indicates that students are acquiring feedback skills and that there is also room for improvement.

Students scored on average insufficient marks on three rubric subitems: structure, applicability, and spelling and grammar. Possibly, these items did not receive enough attention during feedback education. The available time for discussing and writing down feedback might be of influence as well. If students experience scarce time, they might rush and neglect some aspects of giving feedback, such as spelling and grammar. Hence, students’ feedback could perhaps be further improved simply by offering more time during the group sessions.

### Strengths and limitation

This study has several strengths. Firstly, the rubric was developed to objectively score feedback quality. This is important as scoring with a rubric is more consistent among researchers than scoring without a rubric.^21^ It was tested in several rounds of pilot runs to ensure uniform scoring among scorers, which was confirmed by similar scores from the independent researcher who was not involved in rubric development. It could therefore become a widely applicable instrument. Another strength is that we had a large database of thematic survey narrative feedback responses from students of all Medicine and Biomedical Sciences undergraduate study years that enabled us to draw accurate conclusions about the average level of narrative feedback quality. A limitation of this study is that there is no golden standard for rubric validation. Studies on methods to develop and validate rubrics were limited. Andrus and colleagues (2018)^27^ and Allen and Knight (2009)^21^ described ways to develop and validate rubrics, and how to increase rubric reliability. According to them, validation requires multiple individuals using the rubric and making sure the scores are consistently independent of the person using the rubric. In our case, both researchers practiced several times during the development of the rubric, after which the rubric was modified to improve consistency and objectivity, and the final rubric was determined. Also, a third independent researcher scored 10% of all feedback answers to make sure the rubric measured what we intended to measure and to assess possible bias.

### Implications for practice and research

Our rubric can help students by being assessed or with self-assessing their feedback and learning on what feedback aspects they can improve. Feedback skills are essential in workplace learning for Medicine and Biomedical Sciences students, as it helps them grow towards the desired performance. Throughout their study, students have to learn to apply their skills in increasingly complex situations. To prepare for master internships and ultimately their job in research or patient care early investment is recommended.^1^^,^^28^^-^^30^ To take full advantage of workplace feedback with new experiences, such as hierarchical relationships, students should have developed skills to give and receive constructive narrative feedback in an undergraduate curriculum. Hence, (peer-assisted-) feedback training and assessment during the undergraduate curriculum is essential. Further research should investigate whether usage of the rubric by students can indeed improve their feedback giving skills and, in a broader sense, their feedback literacy. This study showed the effect of the effort of training of narrative feedback skills during the undergraduate program and the improvement of feedback quality. So, besides the microlevel (student level) of feedback skills, the developed instrument is also an instrument to measure quality of training on a macro (organisational) level, valuable for educators for continuous curriculum improvement.

## Conclusions

We present a useful and reliable instrument to measure feedback quality by teachers as well as in a self-assessment setting. This rubric can facilitate and stimulate the development of appropriate narrative feedback skills. When translating to other curricula, we recommend to take into account the adaptation of generic content in the local setting. The rubric, together with examples of good and poor narrative feedback, can be used to further enhance education on feedback given to students.

### Acknowledgements

We thank Dr Thomas Hoogeboom for his help with the study design and Dr Florieke Eggermont for being our independent researcher.

### Conflict of Interest

The authors declare that they have no conflict of interest.
